# OGG1 activation improves T cell resilience to oxidative stress after allo-SCT and T cell engager exposure

**DOI:** 10.1038/s41375-025-02783-4

**Published:** 2025-10-15

**Authors:** D. Saul, C. Lischer, H. Bruns, N. Ziegler, A. Kannt, M. Michel, D. Mougiakakos

**Affiliations:** 1 Department for Hematology, Oncology and Cell Therapy, Otto-von-Guericke University, Magdeburg, Germany; 2 Department of Medicine 5, Hematology and Oncology, Friedrich-Alexander-Universität Erlangen-Nürnberg and University Hospital Erlangen, Erlangen, Germany; 3 Fraunhofer Institute for Translational Medicine and Pharmacology (ITMP), Frankfurt, Germany; 4 Fraunhofer Cluster of Excellence for Immune Mediated Diseases (CIMD), Frankfurt, Germany; 5 Institute of Clinical Pharmacology, Faculty of Medicine, Goethe University Frankfurt, Frankfurt, Germany; 6 Department of Oncology and Pathology, Science for Life Laboratory and Center for Molecular Medicine, Karolinska Institute and Karolinska Hospital, Stockholm, Sweden; 7 Healthcampus Immunology, Inflammation and Infectiology (GC-I3), Otto-von-Guericke-University, Magdeburg, Germany

**Keywords:** Translational research, Transplant immunology

## Abstract

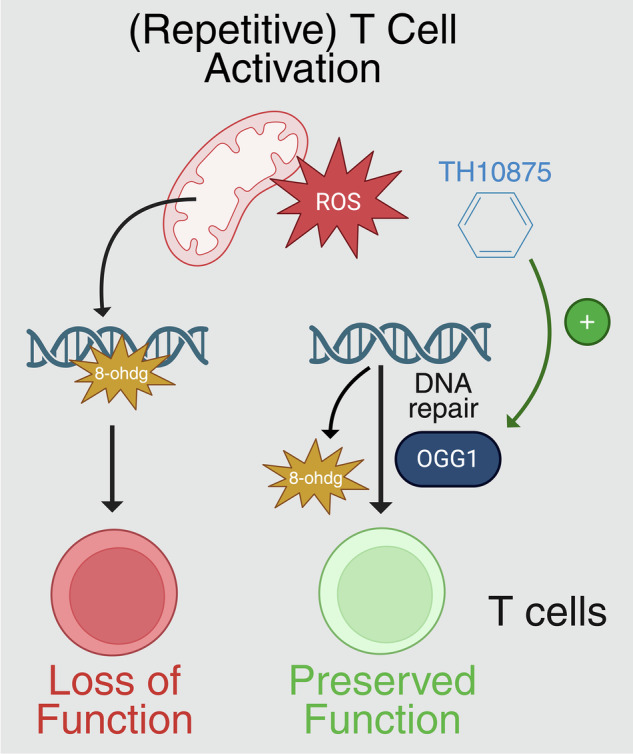

## To the Editor,

Relapse remains the major cause of treatment failure after allogeneic stem cell transplantation (allo-SCT), often related to impaired immune surveillance by donor-derived T cells. Oxidative stress is a hallmark of allo-SCT leading to accumulation of oxidized DNA lesions, most notably 8-hydroxy-2’-deoxyguanosine (8-ohdg) in reconstituting T cells [1]. High 8-ohdg level correlated with impaired functionality and increased relapse risk [1]. The clinical success of histamine dihydrochloride, an antioxidant agent, in combination with interleukin-2 for relapse prevention in AML highlights the therapeutic relevance of redox modulation to restore T and NK cell functionality [2]. Sustained stimulation of the T cell receptor (TCR) in alloreactive T cells results in mitochondrial hyperreactivity and abundant superoxide generation [3]. Oxidized bases lead to replication stress and double-strand breaks, which not only impair gene expression but also induce premature senescence, a state that limits efficacy of T cell-based therapies. T cell fitness, which encompasses metabolic resilience and adaptability, has emerged as a key determinant of successful immune reconstitution and therapeutic efficacy [4]. In fact, DNA integrity, particularly resistance to oxidative DNA damage, is an underappreciated facet of T cell fitness, as DNA damage accelerates T cell aging [5]. In this context, “DNA fitness” emerges as a potential prerequisite for sustained T cell function.

The DNA-glycosylase OGG1 is the key enzyme initiating base excision repair of 8-ohdg, thereby maintaining genomic stability [6]. Recent work has identified TH10785, a small-molecule OGG1 activator, which enhances its repair activity [7]. Therapeutic benefit of OGG1 activation has already been demonstrated in preclinical models, where it improved tissue function [8]. Here, we explore the potential of OGG1 activation as a strategy to restore and preserve T cell function under oxidative stress. By improving DNA repair capacity, we aim to improve T cell-based therapies.

## Material and methods

### Ethics approval

All methods were performed in accordance with the relevant guidelines and regulations, including the Declaration of Helsinki. Peripheral blood samples were retrieved from patients and healthy donors (HD) upon informed consent. Ethics approval was obtained from the local ethics committees of Friedrich-Alexander University Erlangen-Nürnberg (approval numbers 200_12, 280_14 B, and 313_17B) and Otto-von-Guericke University Magdeburg (approval number 61/22). Written informed consent was obtained from all participants.

### Cells

Peripheral blood mononuclear cells (PBMCs) were isolated using Ficoll-Paque (PAN-biotech, Germany). HL-60 and Nalm-6 cells were obtained from DSMZ (Germany).

### Flow cytometry

For flow cytometry (FACS) analyses, samples were stained with fluorochrome-conjugated antibodies and dyes (Supplementary Table 1) on a FACS Canto II flow cytometer (BD Biosciences, NJ, USA) or on a Cytek NL-3000 full spectrum flow cytometer (Cytek Biosciences, CA, USA). Data was analyzed using FlowJo Version 10 (FlowJo LLC, OR, USA).

For more information, see the Supplementary Information.

## Results and discussion

**Fig. 1:**
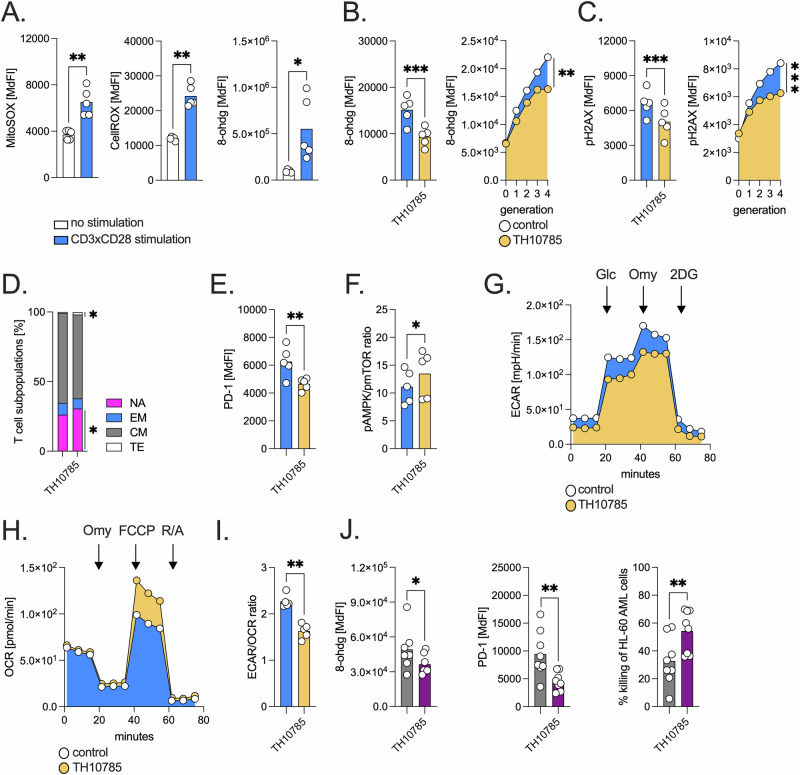
Activation-induced oxidative DNA damage compromises T cell function and metabolic fitness, rescued by OGG1 activation. T cells were stimulated using anti-CD2-/CD3/-CD28 beads throughout all experiments. Where indicated, cells were treated with the OGG1 activator TH10785 or the corresponding DMSO vehicle control. **A** T cells from healthy donors (HDs, *n* = 5) were cultured ± activating beads. Mitochondrial ( = MitoSOX) and total cellular ROS ( = CellROX) as well as 8-ohdg formation were semi-quantified by flow cytometry (FACS) based on the median fluorescence index (MdFI). **B** T cells of HDs (*n* = 5) were stimulated ± TH10785. The left panel shows the 8-ohdg content in the T cells and the right panel shows the content in the different T cell generations as measured by FACS and based on the MdFI. **C** T cells of HDs (*n* = 5) were stimulated ± TH10785. The left panel shows the pH2AX content in the T cells and the right panel shows the content in the different T cell generations as measured by FACS and based on the MdFI. **D** In activated T cells of HDs (*n* = 6) that were cultured ± TH10875, proportion of naïve (NA), central memory (CM), effector memory (EM), and terminally differentiated effector memory (TE) T cells was determined after 96 h by FACS. **E** T cells from HDs (*n* = 5) were stimulated ± TH10785 and PD-1 levels assessed by FACS. **F** T cells from HDs (*n* = 5) were activated ± TH10785. The ratio of phosphorylated AMPK (pAMPK) to phosphorylated mTOR (pMTOR) was analyzed by FACS. **G** Metabolic flux analysis (Seahorse XFe96) was performed in activated T cells ± TH10785 from HDs (*n* = 4), assessing glycolytic (extracellular acidification rate, ECAR) and **H** mitochondrial parameters (oxygen consumption rate, OCR) under basal conditions and after sequential injection of glucose (Glc), oligomycin (Omy), carbonyl cyanide-p-trifluoromethoxyphenylhydrazone (FCCP), 2-deoxy-D-glucose (2-DG), and rotenone/antimycin A (R/A). **I** The ECAR/OCR ratio of activated T cells ± TH10785 from HDs (*n* = 5) was calculated as a surrogate for glycolytic versus oxidative metabolism. **J** T cells from allo-SCT patients (*n* = 8, between day +30 to +60 after allo-SCT) were stimulated ± TH10785, followed by analysis of 8-OHdG level, PD-1 expression, and their AML cell killing capacity in the presence of CD33xCD3 T cell-engaging antibodies. ‘n’ indicates the number of individual donors or patients; *P* value: *P < 0.05; **P < 0.01; ***P < 0.001.

First, we showed in healthy donor T cells that production of mitochondrial and total cellular ROS in response to TCR activation is associated with 8-ohdg formation (Fig. 1A). The highest ROS and 8-ohdg accumulation were seen in the most activated T cells co-expressing CD25 and CD69 (Supplementary Fig. 1A). We also observed progressive 8-ohdg accumulation with increasing T-cell proliferation. The highest 8-ohdg levels were detected during the S phase of the cell cycle, consistent with the known vulnerability of replicating DNA (Supplementary Fig. 1B, C). Central memory (CM) T cells were the most affected subset (Supplementary Fig. 1D). This is of particular interest, as CM T cells are key players in immunotherapy because of rapid antigen responses, high proliferative capacity, superior persistence, and potential to differentiate into effector cells [9]. Despite the pronounced oxidative DNA damage, OGG1 expression levels were not reduced in CM T cells (Supplementary Fig. 1E).

OGG1 plays a critical role in repairing 8-ohdg lesions [6]. To investigate its functional relevance in T cells, we screened 45 healthy donors for the known OGG1-Ser326Cys polymorphism. This polymorphism has been associated with impaired DNA repair [10]. Three donors carried the biallelic variant (Supplementary Fig. 1F). After stimulation, T cells from these individuals showed higher 8-ohdg levels and increased H2AX phosphorylation (pH2AX), an early marker of the DNA damage response (DDR) (Supplementary Fig. 1G). However, due to the low frequency of homozygous OGG1-Ser326Cys carriers in European populations, we do not consider this polymorphism to be a major contributor to the differential 8-ohdg accumulation in reconstituting T cells after allo-SCT in our patient cohorts [1]. Notably, frequency of homozygous carriers is higher in East Asian populations, which might necessitate region-specific considerations in future studies.

TH10785, has recently been identified as a specific OGG1 activator [7]. Treatment with TH10785 reduced activation-induced 8-ohdg and pH2AX accumulation (Fig. 1B, C, Supplementary Fig. 2A). This effect was also observed in T cells carrying the biallelic OGG1-Ser326Cys polymorphism (Supplementary Fig. 2B). In our short-term T cell activation assays, TH10785 led to enrichment of naïve T cells and a notable reduction in PD-1 expression, which is associated with T cell exhaustion, elevated 8-ohdg levels, and increased relapse risk following allo-SCT [1] (Fig. 1D, E).

Metabolic fitness is a critical determinant of T cell functionality [4]. Treatment of activated T cells with TH10785 resulted in a shift of AMP-activated protein kinase (AMPK) and of mechanistic target of rapamycin (mTOR) signaling towards AMPK (Fig. 1F). This is in line with previous reports suggesting that mTOR inhibition or enhancement of AMPK signaling can improve T cell function [11]. Concomitantly, we observed upregulation of CD36, a fatty acid transporter, but not of glucose transporter 1 indicating altered substrate utilization (Supplementary Fig. 2C). Metabolic flux analysis revealed a reduction in basal and reserve glycolytic activity and an increase in mitochondrial respiratory reserve, which together indicate a metabolic shift away from aerobic glycolysis and towards oxidative phosphorylation (Fig. 1G–I, Supplementary Fig. 2D, E). Increased mitochondrial fitness has been associated with improved antitumor efficacy of T cells, and we observed increased expression of the activation markers CD69 and CD137 [12] (Supplementary Fig. 2F). However, in vitro elimination of AML cells in presence of bispecific CD33xCD3 antibodies was not improved (Supplementary Fig. 2G). We then repeated the experiment using T cells from patients 30–60 days after allo-SCT. In these cells, TH10785 treatment again reduced 8-ohdg levels and PD-1 expression. Importantly, in contrast to healthy donor T cells, TH10785-treated post-allo-SCT T cells showed enhanced cytotoxic activity against AML targets (Fig. 1J). This enhanced killing capacity may reflect the increased baseline activation status and stress load of reconstituting T cells in the early post-transplant period compared to short-term stimulated healthy donor cells [1], which is further supported by the fact that less stressed, healthy donor-derived T cells display a higher cytotoxic activity against AML cells (Supplementary Fig. 2H). Interestingly. baseline 8-OHdG levels in patient-derived T cells negatively correlated with the relative increase in HL-60 killing capacity upon TH10785 treatment, suggesting that a lower oxidative DNA damage burden may predict greater functional rescue (Supplementary Fig. 2I).

To further investigate the impact of oxidative DNA damage under T-cell exhausting conditions, we established a model of chronic T-cell stimulation. Clinical treatment regimens differ in their exposure kinetics: for example, blinatumomab, a CD19×CD3 bispecific T cell engager, is administered as a continuous intravenous infusion over 28 days per cycle and has been shown to induce progressive T-cell exhaustion during ongoing exposure—a process that, in the same study, could be mitigated by introducing treatment-free intervals [13]. Nonetheless, even when bispecific antibodies are administered at longer dosing intervals, functional decline of T cells may still occur with increasing treatment cycles [14]. Our model therefore represents a stringent, yet clinically relevant, setting of repeated antigen engagement. In our model, repeated stimulation with CD19xCD3 bispecific antibodies in presence of CD19⁺ target cells led to progressive 8-ohdg accumulation (especially in CM T cells), activation of H2AX, and increased PD-1 expression (Supplementary Fig. 3A, B). In parallel, we observed a gradual decline in both proliferative capacity and cytokine production with each subsequent stimulation, consistent with developing functional exhaustion (Supplementary Fig. 3C).

**Fig. 2:**
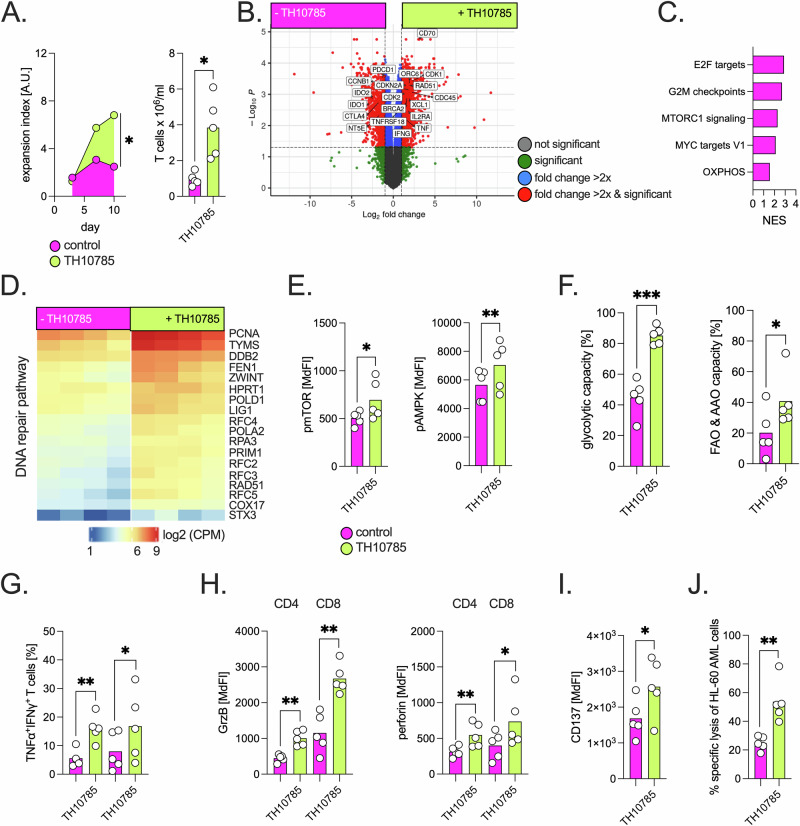
OGG1 activation prevents exhaustion in repetitively stimulated T cells. **A** T cells from HDs (*n* = 5) were repetitively stimulated on days 0, 3, and 7 via co-culture with Nalm-6 cells and bispecific CD19xCD3 antibodies ( ± TH10785). Shown are the T cell expansion index (days 3, 7, and 10) and absolute T cell counts on day 10. **B** RNA sequencing was performed on repetitively stimulated T cells from HDs (*n* = 4) at day 10. The volcano plot shows differentially expressed genes in TH10785-treated versus untreated T cells; selected upregulated genes (adjusted *p* < 0.05, log₂ fold change >2) are annotated. **C** Gene set enrichment analysis (GSEA) reveals pathways enriched in TH10785-treated HD-derived T cells (*n* = 4). GSEA was performed using the human version of the Molecular Signatures Database’s (MSigDB) “hallmark gene sets”. Bar length indicates normalized enrichment score (NES). **D** The heatmap shows differentially expressed DNA repair-related genes (adjusted *p* < 0.05; log₂ fold change > 1) in the T cells from HDs (*n* = 4). Rows represent log₂-transformed, count per million (CPM)-normalized expression values. On day 10, metabolically profiled HD-derived T cells (*n* = 5) showed (**E**) altered phosphorylation levels of AMPK and mTOR and **F** changes in glycolytic and mitochondrial fatty acid/amino acid oxidation capacity (FAO/AAO), as measured by SCENITH. **G** Polyfunctional TNFα⁺IFNγ⁺ T cells were quantified by FACS. **H** Expression of granzyme B and perforin, **I** expression of CD137, and **J** AML cell lysis capacity in the presence of CD33xCD3 bispecific antibodies were analyzed in T cells from HDs (*n* = 5) on day 10. ‘n’ indicates the number of individual donors or patients; *P* value: *P < 0.05; **P < 0.01; ***P < 0.001.

Pharmacological OGG1 activation improved the proliferative potential of repetitively activated T cells (Fig. 2A). As previously shown, 8-ohdG levels increased in parallel with cell division; however, TH10785-treated T cells exhibited lower 8-ohdg accumulation relative to their proliferation rate, indicating more effective DNA repair during expansion (Supplementary Fig. 3D). TH10785 treatment resulted in a shift towards CD4⁺ CM T cells (Supplementary Fig. 3E, F). Recently, we and others have demonstrated that CD4⁺ T cells can contribute substantially to antitumor immune responses, not only through helper functions but also via direct cytotoxic activity [15]. On day 10, we performed bulk RNA sequencing (GSE309061) and found that T cells clustered distinctly according to treatment condition (Supplementary Fig. 3G). Differential gene expression analysis revealed higher levels of key functional genes, such as *IL2RA*, *TNF*, and *IFNG*, in TH10785-treated cells (Fig. 2B). Notably, gene sets previously identified by Philipp et al. to be enriched after resting periods in repetitively stimulated T cells, indicating improved metabolic fitness and proliferative potential, were also enriched in TH10785-treated cells, despite the absence of a treatment pause (Fig. 2C) [13]. This suggests that OGG1 activation may mimic aspects of functional recovery. Moreover, TH10785 induced broad activation of DDR pathways, pointing to a “functional spreading” effect beyond base excision repair (Fig. 2D). While the underlying mechanisms remain to be fully understood, accumulating evidence suggests that OGG1 may also participate in gene regulation, potentially through chromatin remodeling or transcriptional control [6]. In line with these transcriptional changes, metabolic signaling and bioenergetic activity were elevated in TH10785-treated T cells (Fig. 2E, F). We confirmed the increase in glycolytic parameters using metabolic flux analyses (Supplementary Fig. 4A), which likely reflects an overall improvement in T cell fitness. The difference from the short-term activation setting, in which glycolytic parameters were reduced (Fig. 1G), may be due to the fact that in the repetitive stimulation model, restored cellular fitness is the predominant driver, whereas in short-term activation, TH10785 treatment may have additionally triggered metabolic reprogramming, an aspect that warrants further investigation.

Effector functions were similarly enhanced, as evidenced by increased IFN-γ and TNF-α production (Fig. 2G, Supplementary Fig. 4B, C). Secretome analyses confirmed a pro-inflammatory cytokine profile, with higher secretion levels of e.g., IL-2, IL-1α, IL-4, and CD70 in the OGG1 activation group. Findings for IL-2, IL-4, and CD70 were confirmed using alternative methods (Supplementary Fig. 4D, E). Finally, we observed an increase in the production of cytotoxic effector molecules (i.e., perforin and granzyme B) (Fig. 2H). Expression of CD137, a marker of activated cytotoxic T cells, upregulated (Fig. 2I). Accordingly, T cells subjected to repetitive stimulation and treated with TH10875 exhibited an enhanced T cell-engager-triggered activity against AML cells (Fig. 2J).

Taken together, pharmacological activation of OGG1 enhances T cells’ DDR, proliferation, metabolic fitness and cytotoxic function, particularly under exhaustive conditions. These findings highlight OGG1 as a promising target for improving T cell functionality under conditions of oxidative stress, such as those observed during allo-SCT or engagement with bispecific antibodies. While the present study does not provide direct evidence for adoptive cell therapies, the observed effects on T cell fitness suggest that OGG1 activation could enhance the efficacy of genetically engineered T cells - a hypothesis that warrants further evaluation.

## Supplementary information

Supplemental Material

## Data Availability

Original data supporting the findings of this study are available from the corresponding author, Dimitrios Mougiakakos (dimitrios.mougiakakos@med.ovgu.de), upon reasonable request.
