# Supplementary material for: OGG1 activation improves T cell resilience to oxidative stress after allo-SCT and T cell engager exposure
Source: Leukemia. 2025 Oct 15;39(12):3037–41. doi: 10.1038/s41375-025-02783-4 (PMC12634423; doi:10.1038/s41375-025-02783-4)
Supplement: Supplementary file 1 — Supplemental Material [file 41375_2025_2783_MOESM1_ESM.pdf]

- Supplemental Information –

Title: OGG1 Activation Improves T Cell Resilience to Oxidative Stress After Allo-SCT and T Cell Engager Exposure

Running Title: Enhancing T Cell Function via OGG1

Authors/Affiliations: Saul D[1], Lischer C[2], Bruns H[2], Ziegler N[3], Kannt A[3,4,5], Michel M[6], Mougiakakos D[1,7]

[1]Department for Hematology, Oncology and Cell Therapy, Otto-von-Guericke University, Magdeburg, Germany.

[2]Department of Medicine 5, Hematology and Oncology, Friedrich-Alexander-Universität Erlangen-Nürnberg and University Hospital Erlangen, Erlangen, Germany.

[3]Fraunhofer Institute for Translational Medicine and Pharmacology (ITMP), Frankfurt, Germany.

[4]Fraunhofer Cluster of Excellence for Immune Mediated Diseases (CIMD), Theodor-Stern-Kai 7, Frankfurt, Germany

[5]Institute of Clinical Pharmacology, Faculty of Medicine, Goethe University Frankfurt, Theodor Stern-Kai 7, Frankfurt, Germany

[6]Department of Oncology and Pathology, Science for Life Laboratory and Center for Molecular Medicine, Karolinska Institute and Karolinska Hospital, Stockholm, Sweden.

[7]Healthcampus Immunology, Inflammation and Infectiology (GC-I, Otto-von-Guericke-University, Magdeburg, Germany.

Supplemental Material and Methods

Cell line authentication

Cell lines were verified by Microsynth AG (Balgach, Switzerland) using their Cell Line Authentication Service. A total of 3 million cells were resuspended in 70% ethanol and shipped to Microsynth for analysis.

Human cell line profiling was performed by assessing highly polymorphic short tandem repeat (STR) loci. STR amplification was carried out using the PowerPlex® 16 HS System (Promega, Madison, WI, USA). Fragment analysis was performed on an ABI 3730xl Genetic Analyzer (Life Technologies, Carlsbad, CA, USA), and resulting STR profiles were analyzed using GeneMarker HID software (SoftGenetics LLC, State College, PA, USA).

T cell stimulation

For short-term stimulation experiments, T cells from healthy donors were isolated by magnetic bead-based negative selection (Miltenyi Biotec) and stimulated using anti-CD2/CD3/CD28-coated microbeads (Miltenyi Biotec) at a bead-to-cell ratio of 1:2. Cells were cultured for 24–96 hours in the presence of either 7.5 µM TH10785 (Selleck Chemicals, TX, USA) or an equivalent volume of DMSO (Carl Roth, Germany) as vehicle control. For intracellular cytokine staining (i.e., TNFα, IFNγ, granzyme B, and perforin), cells were restimulated with 50 nM phorbol 12-myristate 13-acetate (PMA, Sigma-Aldrich, MO, USA) and 1 µM ionomycin (Calbiochem, Germany) for 4 hours in the presence of GolgiPlug and GolgiStop (BD Biosciences, NJ, USA) for the final 3 hours.

For long-term stimulation experiments, T cells from healthy donors were isolated as described above and co-cultured with Nalm-6 cells at an effector-to-target (E:T) ratio of 2:1 in the presence of 100 pmol bispecific anti-CD19xCD3 antibody (hCD19xCD3, InvivoGen, CA, USA). Cultures were treated with 7.5 µM TH10785 or DMSO. An irrelevant bispecific antibody (βGalxCD3, InvivoGen) served as a control. On days 3 and 7, cultures were refreshed by replacing medium, re-adding Nalm-6 cells, hCD19xCD3, and respective treatments. Intracellular cytokine and cytotoxic molecule staining was performed as described above following PMA/ionomycin restimulation.

Cell cycle analysis

T cells were stained using the Apoptosis, DNA Damage, and Cell Proliferation Kit (BD Biosciences) following a 1-hour incubation with 10 µM BrdU, according to the manufacturer's instructions.

T cell proliferation assay (and 8-OHdG co-staining)

T cells were labeled with violet proliferation dye 450 (VPD450; BD Biosciences) according to the manufacturer's protocol and subsequently stimulated with anti-CD2/CD3/CD28-coated microbeads (Miltenyi Biotec). After the indicated time points, cells were harvested, washed, and fixed using Fix Buffer I (BD Biosciences). This was followed by permeabilization with Perm Buffer III (BD Biosciences). Intracellular staining for oxidative DNA damage was performed using an 8-OHdG antibody (clone 15A3, Abcam, #ab48508) for 30 minutes at 4°C temperature in the dark.

Metabolic flux analysis

Metabolic flux analyses were performed on the Seahorse XFe 96 (Agilent, CA, USA), as previously described in detail[1]. Data was analyzed using Seahorse Wave Software 2.6.3 and parameters were calculated with Microsoft Excel Version 16 (Microsoft, WA, USA).

Calcein release assay

Target cells were labeled with Calcein-AM (Thermo Fisher Scientific, MA; USA) and co-cultured with T cells at an effector-to-target (E:T) ratio of 10:1 in the presence of 100 pmol bispecific CD3-targeting antibody. After 3 hours of incubation at 37 °C, supernatants were collected and transferred to a new plate for fluorescence measurement (excitation: 485 nm; emission: 535 nm). Spontaneous calcein release from target cells alone was subtracted from all samples. Maximum lysis was determined by treating target cells with 1% Triton X-100. Specific lysis was calculated by comparing fluorescence values between control and treatment conditions with the bispecific antibody.

Enzyme-linked immunosorbent assay (ELISA)

ELISA for IFNγ, IL-2, and TNFα (from Biolegend, CA, USA) was performed on culture supernatants collected at the indicated time points using a SpectraMax M3 microplate reader (Molecular Devices, CA, USA), following the manufacturer's instructions.

FACS-based functional metabolic profiling (SCENITH)

FACS–based metabolic profiling was performed using an adapted version of the SCENITH protocol[2]. Briefly, cells were treated with 10 µg/mL puromycin and incubated under the following conditions: untreated (control), in the presence of 2-deoxy-D-glucose (2-DG, 100 mM), oligomycin (1 µM), or both inhibitors combined. Following treatment, $CD3^+$ T cells were identified by surface staining, and puromycin incorporation was detected using an anti-puromycin antibody after fixation and permeabilization with the Cytofix/Cytoperm buffer (BD Biosciences), according to the manufacturer's instructions.

Metabolic capacities for glycolysis, fatty acid oxidation (FAO), and amino acid oxidation (AAO) were calculated based on the median fluorescence intensity of the puromycin signal under different conditions as follows:

Glycolytic capacity (in %) = 100 - [100 x ((untreated - oligomycin) / (untreated - 2-DG & oligomycin)]
FAO&AAO capacity (in%) = 100 - [100 x ((untreated - 2-DG) / (untreated- 2-DG & oligomycin)]

Reactive oxygen species

Mitochondrial superoxides and total cellular reactive oxygen species (ROS) were determined by FACS using MitoSOX™ Red and CellROX™ Deep Red (Thermo Fisher Scientific).

Genotyping of OGG1 polymorphism

Genomic DNA was isolated from PBMCs using the QIAamp Blood Mini Kit (Qiagen), following the manufacturer's protocol. PCR amplification was performed using 10 µM of each primer specific for the wild-type (Ser) and mutant (Cys) alleles of the OGG1 gene. For detection of the wild-type OGG1 allele (Ser326), PCR yielded bands of 252 bp and 406 bp, whereas the mutant allele (Cys326) generated bands of 194 bp and 406 bp. PCR products were separated by agarose gel electrophoresis and visualized using ethidium bromide under UV illumination.

Proteomic measurement

Supernatants from long-term T cell cultures were collected on day 10. The Olink Target 96 Immuno-Oncology Panel (Olink Proteomics, Uppsala, Sweden) was used to quantify 92 proteins in the culture supernatants. Protein measurements were performed using the Proximity Extension Assay (PEA) technology, according to the manufacturer's protocol.

RNA sequencing

On day 10, T cells were harvested and residual Nalm6 cells were depleted using anti-CD10 microbeads (Miltenyi Biotec). T cells were lysed, and total RNA was extracted using the innuPREP DNA/RNA Mini Kit (Analytik Jena AG, Jena, Germany), including an on-column DNase digestion step (RNase-Free DNase Set, Qiagen) to remove residual genomic DNA. RNA concentration and purity were assessed using the NanoDrop™ Lite spectrophotometer (Thermo Fisher Scientific). Samples were diluted in RNase-free water to a final concentration of ≤200 ng/µL. RNA sequencing was performed by Eurofins/GATC Biotech (INVIEW Transcriptome Discover service). A strand-specific cDNA library was prepared, and sequencing was conducted on an Illumina platform, generating ≥30 million paired-end reads per sample (2 × 150 bp read length).

Data analysis

Differential gene expression analysis was performed using both DESeq2 and edgeR to ensure robust identification of significantly regulated genes. Gene Set Enrichment Analysis (GSEA) was carried out using the human "Hallmark" gene sets from the Molecular Signatures Database (MSigDB). Enriched pathways were manually curated based on their biological relevance, and enrichment was quantified using normalized enrichment scores (NES). Differentially expressed DNA repair–related genes (adjusted $p < 0.05$; $|\log_2$ fold change$| > 1$) were visualized in a heatmap displaying $\log_2$-transformed, CPM-normalized expression values.

Statistics

Outliers were identified using the ROUT method ($Q = 1\%$). Normality was assessed via Shapiro-Wilk and Kolmogorov-Smirnov tests. Parametric tests (unpaired/paired t-test, one-way or repeated-measures ANOVA) were used for normally distributed data; nonparametric tests (Mann-Whitney, Wilcoxon, Kruskal-Wallis, Friedman) were applied otherwise. Analyses were performed in GraphPad Prism (v10); $p < 0.05$ was considered significant.

Supplemental Table 1: Antibody list for multiparametric flow cytometry.

Antigen	Fluorochrome	Clone	Isotype	Identifier	Company
AMPKα (pThr172)	FITC	Polyclonal	Rabbit IgG	orb8540	Biorbyt
CD3	FITC	37895	Mouse IgG2a, κ	317306	Biolegend
	APC/Cy7	HIT3a	Mouse IgG2a, k	300318	Biolegend
	APC Fire 810	SK7	Mouse IgG1, κ	344858	Biolegend
	PerCP/Cy5.5	OKT3	Mouse IgG2a, k	317336	Biolegend
CD4	FITC	OKT4	Mouse IgG2b, κ	317408	Biolegend
	BV 510	OKT-4	Mouse IgG2b, κ	317444	Biolegend
	BV 605	RPA-T4	Mouse IgG1, κ	562658	BD Biosciences
	BV 605	SK3	Mouse IgG1, κ	562658	BD Biosciences
	PB	OKT4	Mouse IgG2b, κ	317429	Biolegend
CD8	APC V 770	REA734	rec. human IgG1	130-110-681	Miltenyi
	APC/Cy7	HIT8a	Mouse IgG1, κ	300926	Biolegend
	BV 711	SK1	Mouse IgG1, κ	563677	BD Biosciences
	FITC	HIT8a	Mouse IgG1, κ	300906	Biolegend
	PerCP/Cy5.5	SK1	Mouse IgG1, κ	344710	Biolegend
	PE/Cy7	SK1	Mouse IgG1, κ	344712	Biolegend
	PE	SK1	Mouse IgG1, κ	344706	Biolegend
CD19	APC	HIB19	Mouse IgG1, κ	982406	Biolegend
	APC/Cy7	SJ25C1	Mouse IgG1, κ	363010	Biolegend
CD25	PE	M-A251	Mouse IgG1, κ	555432	BD Biosciences
CD36	FITC	5-271	Mouse IgG2a, κ	336204	Biolegend
CD45RA	BV 570	HI100	Mouse IgG2b, κ	304132	Biolegend
CD45RO	PE/Cy7	UCHL1	Mouse IgG2a, κ	304230	Biolegend
CD69	BV 650	FN50	Mouse IgG1, κ	310934	Biolegend
	PerCP/Cy5.5	FN50	Mouse IgG1, κ	310926	Biolegend
CD70	PerCP Cy5.5	113-16	Mouse IgG1, κ	355108	Biolegend
CD137 (4-1BB)	PE/Cy7	4B4-1	Mouse IgG1, k	309818	Biolegend
CD152 (CTLA-4)	PE	L3D10	Mouse IgG1, κ	349906	Biolegend
CD197 (CCR7)	AF 647	G043H7	Mouse IgGa, κ	353218	Biolegend
	BV 650	G043H7	Mouse IgG2a, κ	353234	Biolegend
CD279 (PD-1)	PE	EH12.2H7	Mouse IgG1, κ	329906	Biolegend
	PE Cy7	EH12.2H7	Mouse IgG1, κ	329918	Biolegend
GLUT1	APC	202915	Mouse IgG2b	FAB1418A	R&D Systems
Granzyme B	Pacific Blue	GB11	Mouse IgG1, κ	515408	Biolegend
IFN-γ	PE/Cy7	4S.B3	Mouse IgG1, κ	502528	Biolegend
IL-4	FITC	MP4-25D2	Rat IgG1, κ	500806	Biolegend
mTOR (pS2448)	PE	O21-404	Mouse IgG1, κ	563489	BD Biosciences
OGG1	PE Cy5.5	2B4	Mouse IgG2b	52724PECY55	novusbio
	-	-	Rabbit polyclonal	PA1-31402	ThermoFisher
Perforin	PE Cy7	B-D48	Mouse IgG1	353315	Biolegend
Puromycin	AF647	12D10	Mouse IgG2a, κ	MABE343	Sigma Aldrich
TNFa	APC	MAb11	Mouse IgG1, κ	502912	Biolegend
H2A.X (pSer139)	PerCP-eF 710	CR55T33	Mouse IgG1, κ	46-9865-42	eBioscience
	PE	CR55T33	Mouse IgG1, κ	12-9865-42	ThermoFisher
secondary ab	AF647	-	Goat polyclonal	ab150079	Abcam
8-OHdG	FITC	15A3	Mouse mAB	ab183393	Abcam
Proliferation Dye	eFluor670	-	-	65-0840-85	eBioscience
Viability	Zombie Aqua	-	-	423102	Biolegend
	Zombie NIR	-	-	423106	Biolegend

Supplemental Figures

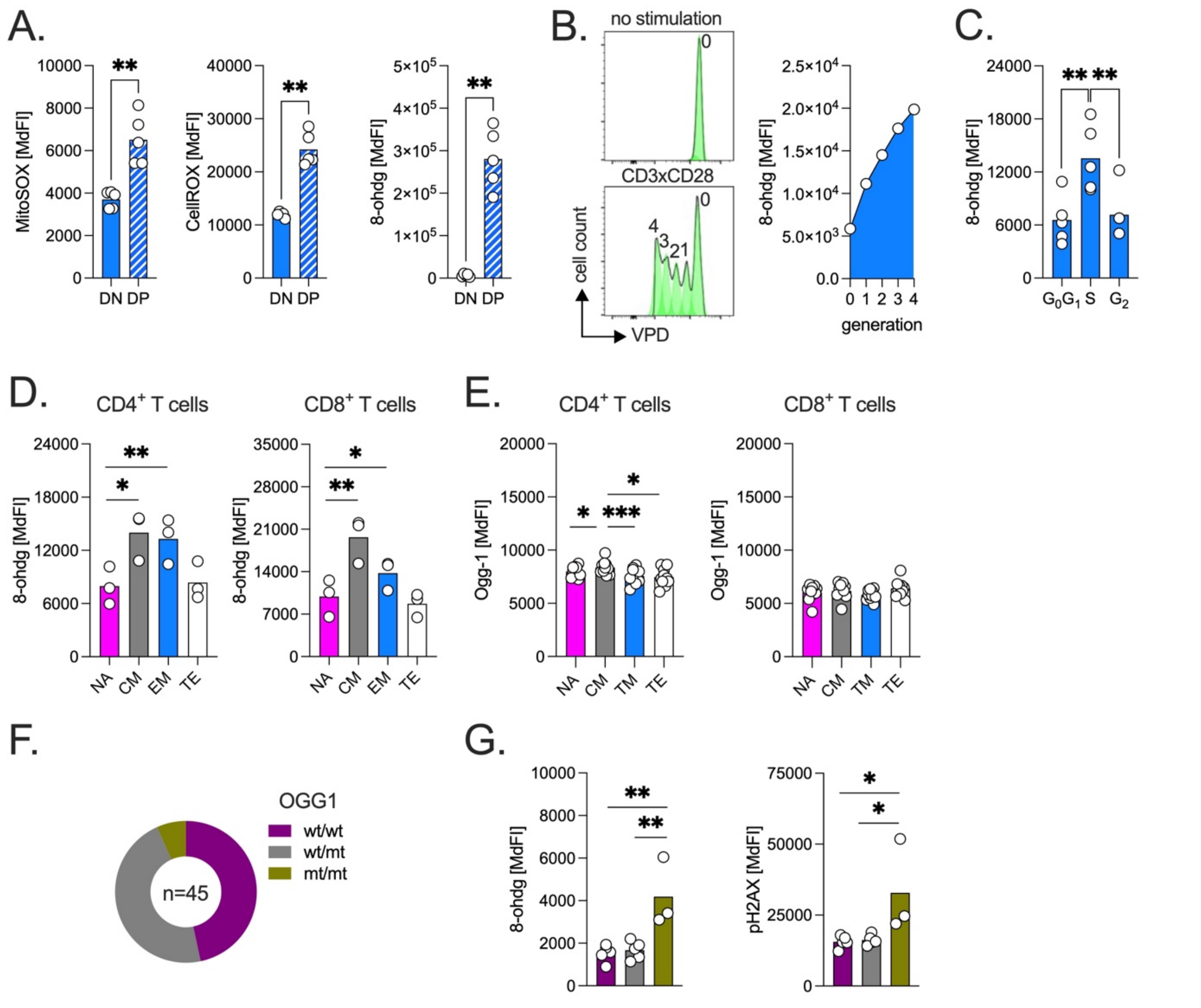

Supplemental Figure 1: (A) Healthy donor-derived T cells (HD, n=5) were cultured in absence/presence of activating anti-CD2/-CD3/-CD28 beads. Mitochondrial (=MitoSOX) and total cellular ROS (=CellROX) as well as 8-ohdg formation were semi-quantified in $CD25^+CD69^+$ double positive (DP) and $CD25^{neg}CD69^{neg}$ double negative (DN) T cells by flow cytometry (FACS) based on the median fluorescence index (MdFI). **(B)** The left panel shows a representative FACS histogram of T cells labeled with violet proliferation dye (VPD) to track cell proliferation. The new generations of T cells formed after stimulation are numbered from 1 to 4. The right panel shows the 8-ohdg content in each generation of stimulated T cells from 5 HDs. **(C)** The level of 8-ohdg was determined in activated T cells of HDs (n=5) in the different phases of cell division (i.e., G_{0-1}, S, and G_2) by FACS. The level of **(D)** 8-ohdg and **(E)** OGG1 were determined in naïve (NA), central memory (CM), effector memory (EM), and terminally differentiated effector memory (TE) $CD4^+$ and $CD8^+$ T cells (n=3 and n=10 HDs respectively) following an anti-CD2/-CD3/-CD28 bead-based stimulation by FACS. **(F)** HDs (n=45) were screened for OGG1-Ser326Cys polymorphisms (mt), which, in a biallelic state (mt/mt), are

associated with reduced DNA repair function. **(G)** HD-derived T cells with OGG1 wt/wt (n=5), wt/mt (n=5), and mt/mt (n=3) were stimulated and induction of DNA oxidation (8-OHdG, left panel) along with subsequent repair processes (pH2AX, right panel) was assessed using FACS. Abbreviations: 'n' indicates the number of individual donors or patients; P value: *P < 0.05; **P < 0.01; ***P < 0.001.

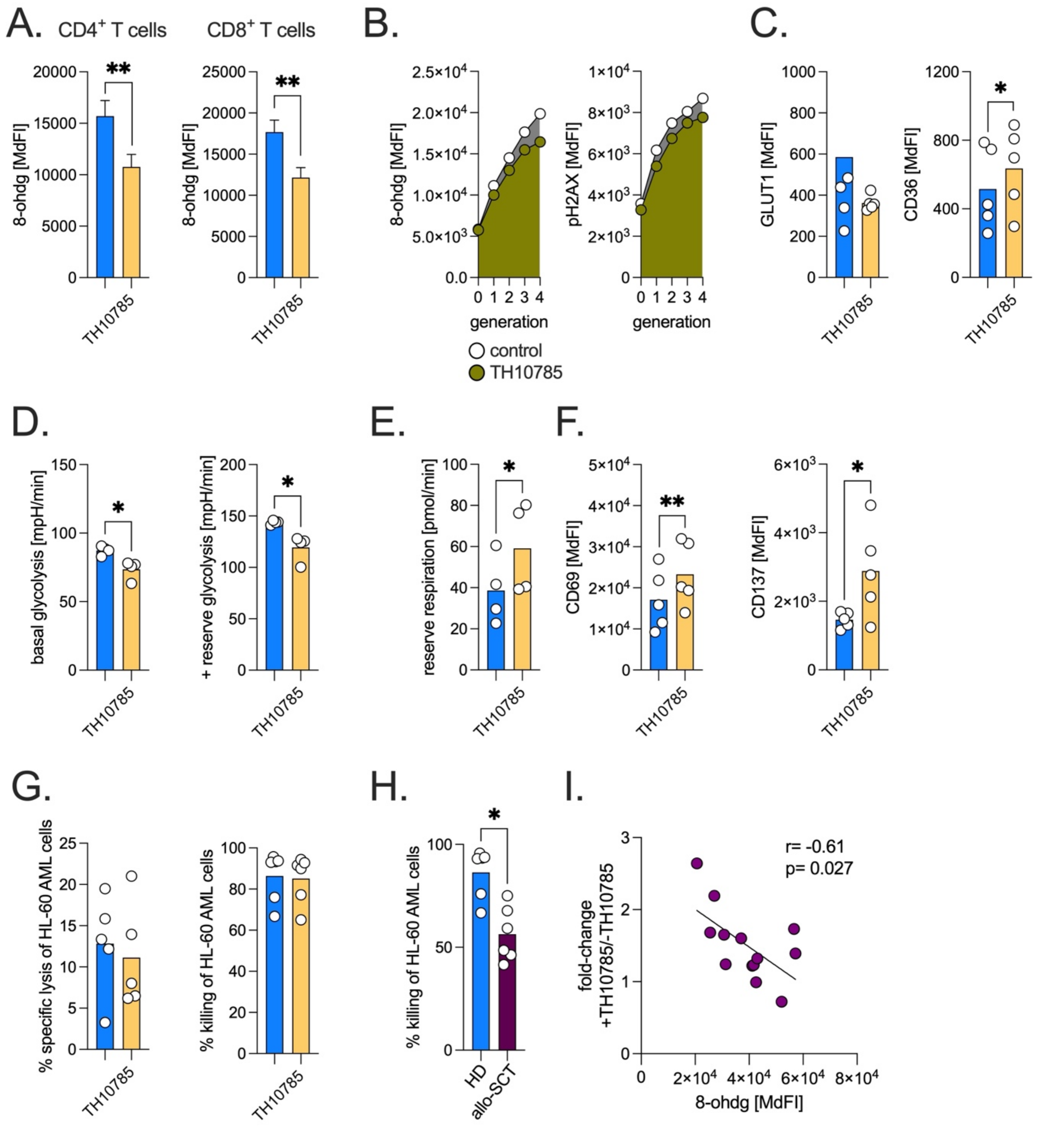

Supplemental Figure 2: (A) T cells from HDs (n=5) were stimulated with anti-CD2/-CD3- and -CD28 beads in the absence/presence of TH10785. The left panel shows the 8-ohdg content in $CD4^+$ T cells and the right panel in $CD8^+$ T cells as measured by FACS and based on the MdFI. **(B)** HD-derived T cells with a biallelic OGG1-Ser326Cys polymorphism (n=2) were stimulated in absence/presence of TH10785. 8-OHdG along with pH2AX MdFI was measured in the different T cell generations using FACS. **(C)** T cells from HDs (n=5) were activated ± TH10785 and surface expression of GLUT1 and CD36 were analyzed by FACS. **(D)** Metabolic flux analysis (Seahorse XFe96) was performed in activated T cells ± TH10785 from HDs (n=4), assessing glycolytic (i.e., basal glycolysis and glycolytic reserve) and (**E)** mitochondrial parameters (i.e., respiratory reserve). **(F)** T cells from HDs (n=5) were stimulated ± TH10785 and

CD69 and CD137 expression assessed. Furthermore, killing capacity of HL-60 AML cells in presence of CD33xCD3 T cell engaging antibodies was analyzed using a **(G)** Calcein release- (left panel) and FACS-based (right panel) experimental approach respectively. **(H)** T cells from HDs (n=6) and allo-SCT patients (n=6) were stimulated with anti-CD2/-CD3/-CD28 beads, followed by analysis of their AML cell killing capacity in the presence of CD33xCD3 T cell-engaging antibodies**.** **(I)** Baseline 8-OHdG levels were determined in T cells from allo-SCT patients (n=13 samples), which were stimulated ± TH10785. Cytotoxic activity against HL-60 AML cells in the presence of CD33×CD3 T cell-engaging antibodies was then assessed. Baseline 8-OHdG levels were correlated with the TH10785-mediated improvement in killing capacity, as shown. Abbreviations: 'n' indicates the number of individual donors or patients; r, Pearson correlation coefficient; P value: *P < 0.05; **P < 0.01; ***P < 0.001.

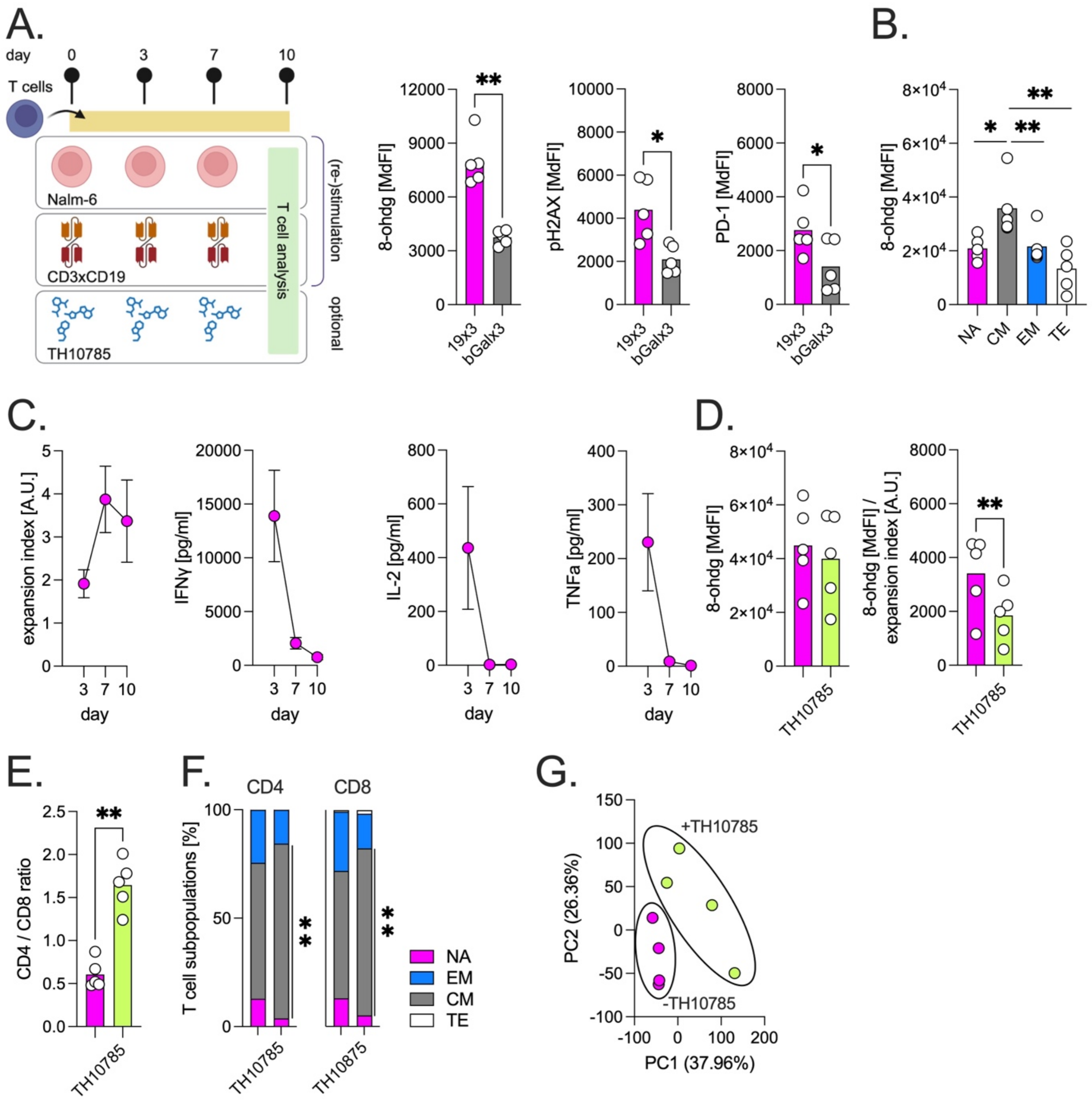

Supplemental Figure 3: (A) In a long-term activation model, T cells of 5 healthy donors (HDs) were repetitively stimulated on days 0, 3, and 7 via co-culture with Nalm-6 cells and CD19xCD3 (19x3) or control bispecific antibodies (βGalxCD3) ± TH10785. On day 10, 8-OHdG, pH2AX, and PD-1 levels were measured in T cells using FACS. **(B)** 8-OHdG content was measured in naïve (NA), central memory (CM), effector memory (EM), and terminally differentiated effector memory (TE) T cells from healthy donors (HDs, n=5) without TH10875. **(C)** The expansion index of VPD-labeled T cells was measured by FACS, and cytokines (IFNγ, IL-2, TNFα) were quantified by ELISA on days 3, 7, and 10 from independent co-culture experiments utilizing T cells from 5 HDs. **(D)** Absolute levels of 8-OHdG (left panel) and the ratio of 8-OHdG to expansion index (right panel) were determined in T cells from HDs (n=5) at day 10. **(E)** The CD4/CD8 ratio and **(F)** distribution of NA, EM, CM, and TE T cells (n=5)

were assessed by FACS. **(G)** The principal component analysis (PCA) of differentially expressed genes in T cells (n=4) at day 10 shows distinct clustering of samples according to TH10875 treatment. Abbreviations: 'n' indicates the number of individual donors or patients; P value: *P < 0.05; **P < 0.01; ***P < 0.001.

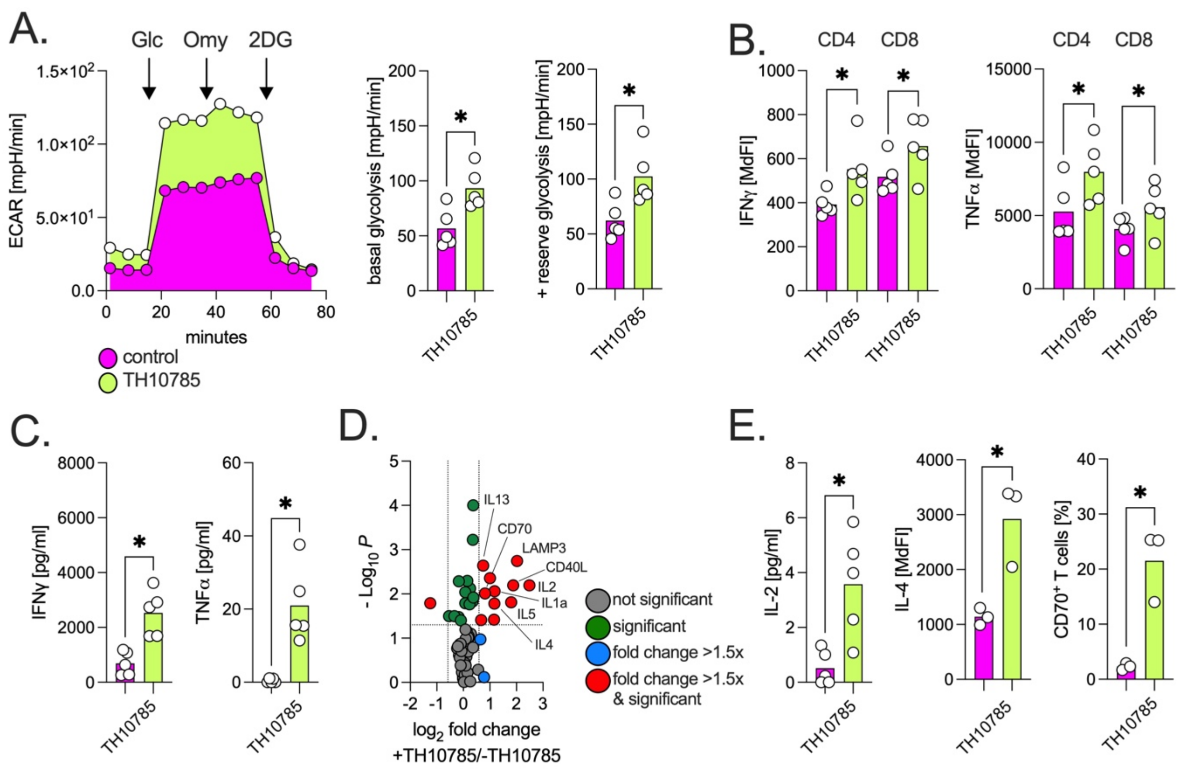

Supplemental Figure 4: (A) Metabolic flux analysis (Seahorse XFe96) was performed in repetitively stimulated T cells ± TH10785 from HDs (n=5), assessing glycolytic parameters (extracellular acidification rate, ECAR) under basal conditions and after sequential injection of glucose (Glc), oligomycin (Omy), and 2-deoxy-D-glucose (2-DG). **(B)** Expression of TNFa and IFNγ was assessed in repetitively stimulated CD4$^+$ and CD8$^+$ T cells ± TH10785 (n=5) by FACS and **(C)** secreted TNFα/IFNγ was quantified in the according supernatants by ELISA. **(D)** Targeted proteomics (i.e., Target 96 Immuno-Oncology panel, Olink) of culture supernatants (day 10) from independent experiments using T cells from six HDs identified differentially secreted proteins; volcano plot highlights proteins significantly enriched in TH10785-treated samples (adjusted p≤0.05, log_2 fold change ≥1.5). **(E)** Secreted IL-2 and IL-4 as well as CD70 expression on T cells from HDs (n=5) were quantified by ELISA and FACS, respectively. Abbreviations: 'n' indicates the number of individual donors or patients; P value: *P < 0.05; **P < 0.01; ***P < 0.001.

Supplemental References

1. Jitschin R, Braun M, Qorraj M, Saul D, Le Blanc K, Zenz T*, et al.* Stromal cell-mediated glycolytic switch in CLL cells involves Notch-c-Myc signaling. *Blood* 2015 May 28; **125**(22)**:** 3432-3436.

2. Arguello RJ, Combes AJ, Char R, Gigan JP, Baaziz AI, Bousiquot E*, et al.* SCENITH: A Flow Cytometry-Based Method to Functionally Profile Energy Metabolism with Single-Cell Resolution. *Cell Metab* 2020 Dec 1; **32**(6)**:** 1063-1075 e1067.
